# Electro-enhanced leaching method for the mobilization of Cr(VI) in contaminated groundwater aquifer

**DOI:** 10.1038/s41598-020-60896-5

**Published:** 2020-03-24

**Authors:** Liyang Hu, Tingting Zhang, Dayi Zhang, Mengyun Jiang, Jie Tan, Jie Li, Zuhong Lin, Zetang Li

**Affiliations:** 1 College of Chemical Engineering, Beijing University of Chemical Technology, Beijing, 100029 People’s Republic of China; 2 School of Environment, Tsinghua University, Beijing, 100084 People’s Republic of China

**Keywords:** Pollution remediation, Environmental impact

## Abstract

Removal of hexavalent chromium [Cr(VI)] from soils and water has been widely studied for its high toxicity. Although leaching method is viewed as an effective approach to eliminate Cr(VI) and some studies attempted to enhance leaching performance via the external electric field, there is little knowledge about the influential factor in electro-leaching system on Cr(VI) removal performance. In this study, an electro-leaching technology was developed for removing Cr(VI) from groundwater aquifer to comprehensively discuss the correlation between the operational parameters and Cr(VI) removal efficiency. When the applied voltage was 20 V and the initial Cr(VI) concentration was 40 mg/kg, Cr(VI) removal efficiency achieved 99.9% in 120 min in the electro-leaching system, 15% higher than the system without the electric field. Cr(VI) removal efficiencies increased with the voltage demonstrating the significant enhancement of the electro-leaching method in removing Cr(VI). When Cr(VI) concentration climbed to 120 mg/kg, Cr(VI) removal efficiency remained above 85%. The effects of different voltages, Cr(VI) concentrations, pollutant distribution and salt content of leaching solution on the leaching effect were also investigated. Meanwhile, the relationship between the current intensity change and the amount of removed Cr(VI) during the electro-leaching process was first investigated, and the relevant model was fitted. There is a quadratic linear correlation between the amount of current change and the amount of removed Cr(VI). This novel electro-enhanced leaching method can effectively remove Cr(VI) from contaminated groundwater aquifer by enhancing the migration of charged contaminant ions during the leaching process, and it is worthy of further study of heavy metal remediation.

## Introduction

Nowadays, heavy metal pollution is getting increasing attention for their inert properties and significant accumulation in organisms through food chain. Either natural process and anthropogenic activities can cause heavy metal pollution, which spread in different media as water, soil and groundwater. Among all heavy metals, hexavalent chromium [Cr(VI)] poses great risks to ecological system and human health due to its high toxicity and mobility^1–3^. Vertical and horizontal migration of Cr(VI) can result in Cr(VI) contamination in groundwater aquifers and expand the polluted area, respectively. As the groundwater aquifer is a saturated soil zone^4^, all the approaches used in soil Cr(VI) decontamination are applicable in groundwater aquifer system, but suffering from the complex aquifer conditions as diverse water chemistry and resistance within the aquifer.

Leaching technology is a promising approach to remove Cr(VI) from soils^5^. Many cases have been reported to effective removal Cr(VI) in soils and groundwater, and the main investigated parameter including the reagents in the processing fluid, the initial concentration of pollutant, coexisting ions in the environment, etc^6,7^. However, traditional leaching technology requires huge amounts regents in leaching solution and does not work well for media with low permeability, restricting its application in practices. Most applied reagents in the processing fluid are organic acids and ethylenediaminetetraacetic acid (EDTA)^8^, which mobilize heavy metals by altering soil acidity, solution ionic strength, redox potential, and complexation^9^. Nevertheless, they are expensive and it is not affordable to use those agents in removing metals in huge amount of soils or groundwater aquifer. Additionally, leaching performance is also dependent on metal migration capacity, which is affected by the permeability of media. For groundwater with low permeability, metal migration takes too long time in traditional leaching system and does not meet the requirement of practical clean up^10^. It is of great urgency to develop approaches to enhance the performance of leaching technology, particularly via reducing the usage of leaching reagents and improving metal migration capability.

The applied electric field strengthen the transportation of metal ions in groundwater and soil, and this electro-migration phenomenon could be useful for the heavy metal remediation in the environment. Applying external electric field are reported to improve Cr(VI) migration capability^11^ and numerous studies suggested satisfactory Cr(VI) removal performance through electrokinetic remediation^12,13^. Zhou *et al*. applied an exchange electrode-electrokinetic remediation using solar energy to clean Cr-contaminated soils and Cr removal efficiency achieved 91.9%^14^. A systematic bench-scale study documented 88.0% Cr removal from abandoned industrial site by applying a voltage gradient of 1 V/cm^15^. Meanwhile, as Cr(VI) has strong solubility, natural groundwater instead of leach solution is possible to elute Cr(VI) from aquifer when its migration capability is strong enough. Although numerous studies proved electric field assisted approach can strengthen leaching technology and focused on the electro-enhanced removal of Cr(VI), few works unraveled the key roles of applied current intensity in the electro-enhanced process.

In this study, we innovated an electro-leaching system in a sand column with groundwater as the leaching solution and an external electric field applied. This method could significantly enhance the electro-migration capacity of metal ions. This system successfully enhanced Cr(VI) removal from groundwater aquifer, and different operational parameters were tested to optimize Cr(VI) removal efficiencies. Our results provided direct evidence that both migration capacity and removal efficiency of Cr(VI) were significant enhanced via electro-leaching under optimal conditions, offering both theoretical and technical support for its future practices in groundwater remediation engineering.

## Materials and Methods

### Materials

Without specifically stated, all the reagents used in this work were of analytical grade. Sands were collected from a local riverbed on April 1, 2018, and passed through a sieve with 2 mm diameter pores. Sands were washed by deionized water to eliminate any possible Cr(VI) contamination. Cr(VI) stock solution (1000 mg/L) was prepared using potassium dichromate (K_2_Cr_2_O_7_).

### Electro-leaching system set-up

**Figure 1:**
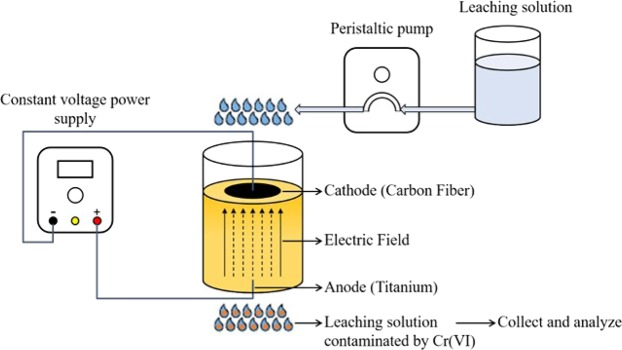
The schematic of the electro-enhanced leaching experiments.

The electro-leaching experiments were carried out in a manufactured system using a cylindrical plastic reactor (6 cm × 6 cm × 9 cm), as illustrated in Fig. 1. At the bottom, one hole (0.5 mm in diameter) was manufactured at the center with another three holes (0.6 mm in diameter) evenly distributed around to allow water leaching through the sand column. A 5-mL syringe was injected at different positions into the sand column to simulate Cr(VI) distribution in groundwater aquifer, filled with Cr (VI) stock solution (1000 mg/L). A carbon fiber was wound into a ring shape and placed in the sand column at 2 cm below the surface as a cathode for the electro-leaching process, whereas a 0.5 mm titanium wire was used as the anode, which was bent into a J shape, protruded into the sand column from the center hole in bottom and placed into the soil column at 0.5 cm to the bottom. A constant voltage power supply (APS3005Si, HUAKO, China) was used to apply an electric field on the whole system. A peristaltic pump (WT600-2J, Longer Pump, USA) injected the leaching solution into the electro-leaching system at a constant flow rate from the top.

### Leaching experiments

In each treatment, 250 g of sands were placed in the system to simulate groundwater aquifers. The leaching process lasted for 120 min and the current intensities were recorded at 0, 10, 20, 30, 40, 50, 60, 90 and 120 min throughout the experiment. The leaching solution was sampled for Cr(VI) concentration measurement and the volume was also measured at the same time interval.

To study the effects of initial concentration and distribution of Cr(VI) in groundwater aquifer on Cr(VI) removal efficiency, different volume of Cr(VI) stock solution was injected to set the initial Cr(VI) in sand column as 20, 40, 60, 80 and 120 mg/kg, respectively. The inject location of Cr(VI) stock solution into the system was adjusted to explore whether Cr(VI) distribution affected leaching performance. Accordingly, the exact inject location was set as 2, 4 and 6 cm from the top, simulating the Cr(VI) plume from top, middle and the bottom layer, respectively.

Both applied voltage and salt content of leaching solution were chosen to investigate the influences of operational parameters on Cr(VI) electro-leaching performance and migration features. The applied voltages were set as 0, 5, 10, 15 and 20 V and the correlation between current intensity and the amount of Cr(VI) was analyzed. The salt content of leaching solution was set as 0, 3.5‰, 7‰, 14‰, 21‰ and the Cr(VI) removal rate under different conditions was investigated.

To verify the effectiveness of electro-enhanced leaching method for actual contaminated groundwater aquifers, we set a series of experiments in a square tank reactor (35 cm × 20 cm × 13 cm). We placed the 4 cm-thick sand column at the bottom of the reactor, and a 1 cm-thick soil column was placed right above it to cover the sand column. A 5-mL syringe was injected at the center of the sand column to simulate Cr(VI) contamination in groundwater aquifer, filled with Cr (VI) stock solution (1000 mg/L). Two strands of carbon fiber were inserted vertically into the sand layer on both sides of the injection point of 4 cm. They were used as cathode and anode for the electro-leaching process. Meanwhile, two hollow fiber membrane bundles were inserted into the sand column at both sides of the reactor near the side wall. One was for injecting and the other was for pumping. We used them to simulate the groundwater runoff in the experiments. A constant voltage power supply (APS3005Si, HUAKO, China) was used to apply an electric field on the whole system. Two peristaltic pumps (WT600-2J, Longer Pump, USA) injected and pumped the leaching solution in the electro-leaching system at a constant flow rate.

### Analytical methods

Cr(VI) concentration in the water before and after adsorption were determined at *λ*_max_ = 540 nm with an ultraviolet-visible spectrophotometer (TU-1901, Beijing Purkinje General Instrument Co., Ltd., China), after complexation with 1,5-diphenylcarbazide. A multimeter (Fluke 175c, USA) was used to measure and record the current intensity and voltage in the electro-leaching system throughout the experiment.

### Data analysis

In each treatment, Cr(VI) removal efficiency was calculated by the following Eq. (1).

1 $${\rm{Removal}}\,{\rm{rate}}=\frac{m}{{m}_{0}}\times 100 \% $$

Here, *m* is the amount of Cr(VI) removed in each treatment and *m*_0_ refers to the initial amount of Cr(VI).

The amounts of electricity generated by current accumulation and Cr(VI) removal were calculated by the following Eqs. (2) and (3).

2 $${Q}_{I}={\int }_{{t}_{1}}^{{t}_{2}}({I}_{t}\times t)$$

3 $${Q}_{Cr}=\frac{m}{52\times 1000}\times 2\times 9.632\times {10}^{4}$$

Here, *Q*_I_ is the amount of electricity generated by current accumulation and *Q*_Cr_ is the amount of electricity generated by Cr(VI) removal. *t* is the different time we chose and *I*_t_ is the current at that moment. *m* is the amount of Cr(VI) removed at the chosen time.

## Results and Discussion

### Influence of electric field on the leaching performance

**Figure 2:**
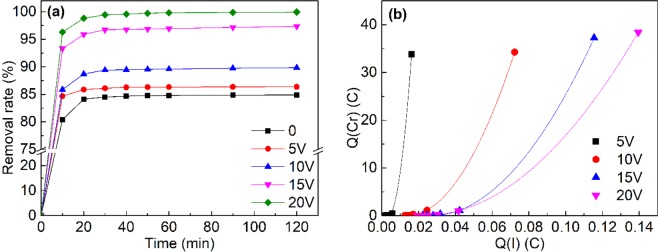
(**a**) Cr(VI) removal efficiency with different applied voltages. (**b**) Correlation of electricity accumulated by current and Cr(VI) removal.

A series of experiments were conducted to study the removal capability with applied electric field. The applied voltage showed significant impacts on Cr(VI) removal efficiency, as illustrated in Fig. 2a. Without the external electric field, Cr(VI) removal efficiency was of 80% in the first 10 min and gradually achieved 85% after 30 min. A similar pattern was also observed for the treatments with the applied voltage that Cr(VI) removal efficiency ranged from 85% (5 V) to 96% (20 V) in the first 10 min. Up to 30 min, Cr(VI) removal capacity reached its peak and the highest Cr(VI) removal efficiency (99%) was achieved in the treatment with 20 V voltage.

Our results proved significant enhancement of Cr(VI) removal in the electro-leaching system. Cr(VI) ion mobilization was promoted by the external electric field and eluted by the leaching solution. The applied voltage is reported to impel cations and metal rich solution to move from anode area towards cathode area through electron-osmosis^15,16^ and alter soil characteristics including pH, moisture, permeability and microflora to dissolve and mobilize the stable metals^17^.

Both the external electric field and the leaching solution contributed to the Cr(VI) removal in the electro-leaching process. To evaluate the contribution of the two impacts factor of the process, the electricity accumulated by current and Cr(VI) removal under different applied voltages were calculated, as shown in Fig. 2b. As the voltage increased, the amount of electricity accumulated by the current was gradually increasing and the electricity accumulated by Cr(VI) removal was also slightly increasing. By polynomial fitting between electricity at different time interval, a certain relationship was revealed. And the R2 value of each polynomial fitting result was above 0.99. This result suggested the Cr(VI) removal amount was also positively correlated with applied voltages. And it can be concluded that the electric current in this system is closely related to the amounts of mobile ions under electromigration^18^. For all of the experiments conducted under constant voltage, the observed electric current also directly reflected the conductivity, resistance, power consumption and transportation phenomena of existing species in electro-enhanced leaching system^19^. Compared with the removal amount in the same period, the decrease of current was caused by the mobilization of Cr(VI). The strengthen of electric field in the sand column would affect the migration capacity of Cr(VI). These two figures supported the conclusion we obtained that the applied voltage could significantly enhanced the Cr(VI) removal capacity of this system. Therefore, the applied voltage was an important factor for the Cr(VI) removal efficiency in the electro-enhanced leaching process.

### Influence of initial concentration on the leaching performance

**Figure 3:**
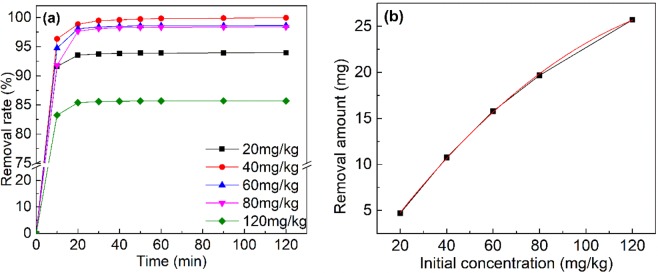
(**a**) The removal rate of Cr(VI). (**b**) The correlativity of removal amount of Cr(VI) and initial concentration of Cr(VI).

The Cr(VI) contamination occurred in the groundwater aquifer normally maintained at a relatively low range. Therefore, a series of experiments were conducted to study the removal capability under different initial Cr(VI) concentration. The initial concentration of Cr(VI) showed significant impacts on Cr(VI) removal efficiency, as illustrated in Fig. 3a. The correlation between the removal amount and the initial concentration of Cr(VI) was also fitting. In the experiment, the initial concentrations of Cr(VI) in the groundwater aquifer were 20/40/60/80/120 mg/kg. The removal amount in the leaching process increased as the elevation of initial concentration. This result indicated that the removal capability of electro-enhanced leaching method varies for different initial concentrations of Cr(VI) in the groundwater aquifer. The outcome of polynomial fitting between different initial concentrations and removal amounts was also shown in Fig. 3b and the R2 is 0.99. The result suggested that the fitting result obtained from Fig. 3b well simulated the removal capacity of the electro-enhanced leaching method under different initial concentration of Cr(VI). The maximum removal rate was 99.9% under 40 mg/kg. 98.4% and 98.6% of Cr(VI) was mobilized under 60 mg/kg and 80 mg/kg. When the concentration reached 120 mg/kg, 85.7% of Cr(VI) was removed. This fitting result would serve a guidebook for the treatment of actual contaminated site using electro-enhanced leaching method. Meanwhile, the electro-enhanced leaching method showed outstanding removal ability at different contamination concentration in the groundwater aquifer.

### Performance of electro-enhanced leaching method under different depth of contaminated layer

**Figure 4:**
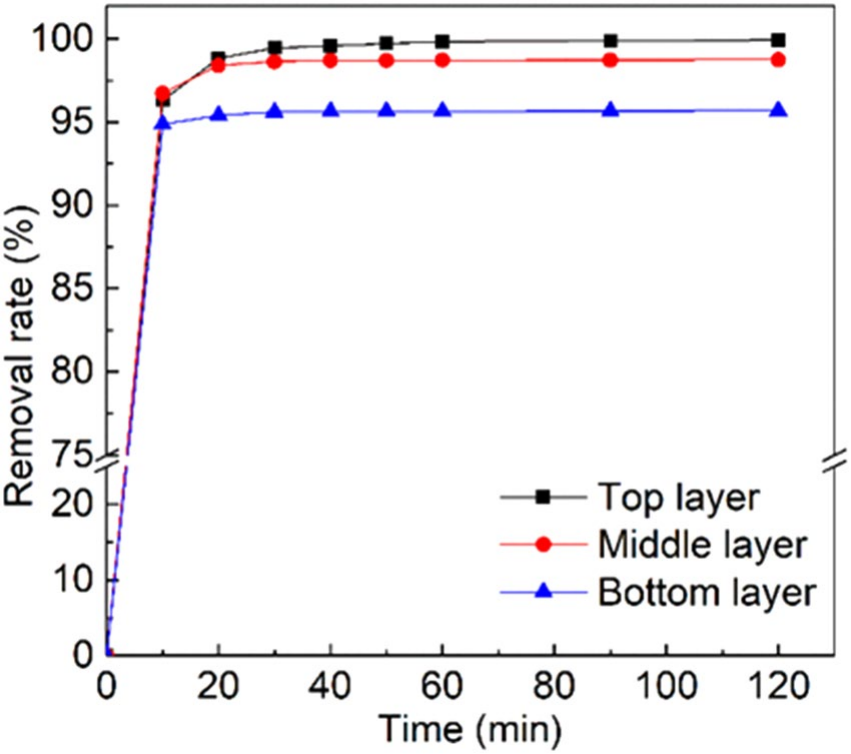
The removal rate of Cr(VI) under different depths of contaminated layer.

In the groundwater aquifer, the pollutant ions tended to migrate and distributed in the different depth of the environment. Different distribution of pollutant in the groundwater aquifer would affect the removal efficiency of electro-enhanced leaching method. In the actual Cr(VI)-contaminated groundwater aquifer, the pollutant tended to migrate from top to bottom under the gravity and rain flushing, and the Cr(VI) in the deep position of groundwater aquifer would cause severe contamination to the groundwater for its high mobility. Szecsody *et al*. investigated the persistence of chromate in vadose zone and aquifer sediments in Hanford, Washington, and the content of Cr(VI) in different depth was also surveyed^20^. This study indicated that the contamination deep down in the groundwater aquifer was still severe. It is quite of great significance to study the removal performance in the different position of aquifer. Figure 4 showed the removal rate of Cr(VI)with different Cr(VI) distribution in the sand column. When the pollutant was placed at the top layer of the sand column, the removal rate was 99.9%. When the pollutants were placed at the middle layer, the removal rate was stable at 98.8%. The removal rate maintained at 95.7% even the Cr(VI) contamination was at the bottom layer. Comparing to the top layer situation, the removal rate was slightly reduced as the contaminants were distributed at the middle and the bottom layers of the sand column. The slight difference in removal capacity was due to the relative position of the contaminants and the electrodes. Based on the distribution of pollutants in the contaminated groundwater aquifer, the electrodes needed to be placed at the proper position to form the electric field for the better enhancement of Cr(VI) removal. By exploring the removal efficiency of different depth contaminants under the treatment of electro-enhanced leaching method, we concluded that this method could effectively remove the Cr(VI) contamination from different depth of groundwater aquifer, and the electro-enhanced leaching method showed great potential in the treatment of the actual complex Cr(VI)-contaminated sites.

### Performance of electro-enhanced leaching method under different salt content of leaching solution

**Figure 5:**
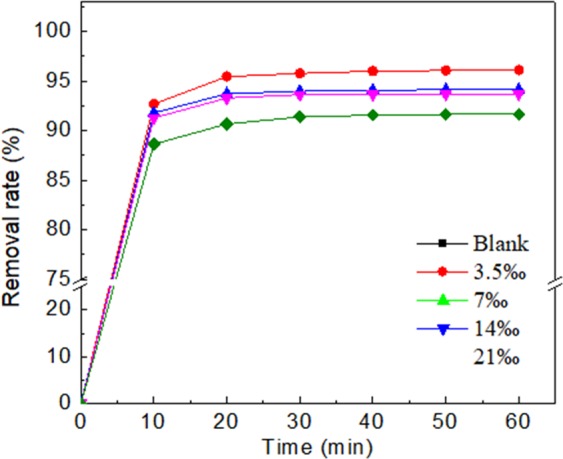
The removal rate of Cr(VI) under different salt contents of leaching solution.

There are numerous kinds of ions coexisting in the groundwater aquifer environment, and salt content is an important characterization index. The salt content of the leaching solution posed significant impact to the strength of electric field in the sand column. The removal efficiency was shown in Fig. 5 as the salt content of the leaching solution changing. As the salt content elevated from 0‰ to 21‰, the removal rate decreased significantly. When the salt content reached 21‰, the removal rate was reduced by 8.3% compared with using deionized water as the leaching solution. This result indicated that the leaching efficiency was suppressed by the salt content of the leaching solution. The salt ions in the leaching solution would affect the dissolution of Cr(VI) in the deionized water during the leaching process. Several studies had found out that the ion competition would affect the adsorption performance^21,22^. We speculated that the ion competition also resulted in the removal capability variation under different salt contents. The salt composition enriched the ion concentration and slightly enhanced the strength of the electric field in the sand column. Therefore, this electro-leaching method was limited in the high salt content aquifer environments.

### Simulated field test in contaminated groundwater aquifer for electro-enhanced leaching method

**Figure 6:**
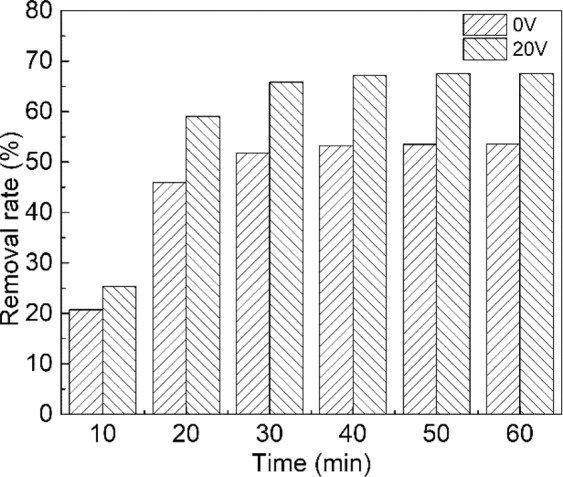
The removal rate of Cr(VI) in the simulated actual contamination of underground aquifers with and without applied voltage.

After conducting series experiments in the reactor, we believed that this electro-leaching method was an effective approach to remove Cr(VI) in the actual Cr(VI)-contaminated underground aquifer. To verify the efficiency of this method in the actual contaminated site, we created a new reactor to simulate the actual contaminated aquifer. Figure 6 showed the removal capacity of electro-leaching method in the simulated scenario. The same tendency was shown comparing with the previous laboratory experiments. When the applied voltage was 20 V, Cr(VI) removal efficiency achieved 67.6% in 60 min in the electro-leaching system, 14.0% higher than the system without the electric field. The electric field significantly improved the leaching efficiency in the experiments. The experiments proved that the electro-enhanced leaching was an effective approach for Cr(VI)-contaminated groundwater aquifer and the same enhancement of Cr(VI) removal by external electric field was also proved. As shown in Table 1, this approach presented by our study is effective and feasible comparing with other soil Cr(VI) pollution treatment methods in the referees.

**Table 1:** Various treatment methods on the remediation of Cr(VI).

Treatment Methods	Cr(VI) concentration *C*_0_ *(mg/kg)*	Removal efficiency *(%)*	Reference
Electrochemistry Flushing & Reduction technology	600	87.5	^23^
Column leaching using EDTA and citric acid	113	75.0	^24^
*Bacillus amyloliquefaciens*	100	70.0	^25^
Nano-FeS coated humic acid complex	100	89.1	^26^
Electro-enhanced leaching	120	85.7	This work

## Conclusion

The Cr(VI) contamination in groundwater aquifer poses a great risk to the groundwater and soil due to the high mobility and toxicity of Cr(VI). Our novel leaching method has proven to be an efficient way to treat the Cr(VI) contamination in groundwater aquifer. The electric field can improve the migration of charged pollutant in groundwater aquifer. Electro-enhanced leaching method was developed by combining the leaching technique and electro-remediation. Artificial contaminated underground aquifer was placed in the reactor. Non-uniform electric field generated by carbon fiber-titanium wire electrode was designed to enhanced the migration of Cr(VI) in the column. The correlation between the current and the removal amount during the leaching process indicated that the removal of Cr(VI) in the sand column was enhanced by leaching and electro-migration. The experiments that conducted under different conditions measured the removal ability of this method, and proved that this method was suitable for a variety of situations. This result will serve as a guide in the practical application of this electro-enhanced leaching process. The novel electro-enhanced leaching method can significantly reduce the Cr(VI) in Cr(VI)-contaminated groundwater aquifer in a relatively short period of time. The removal ability and electro-migration capacity was evaluated. The electro-enhanced leaching method is a promising and novel treatment method that could achieve thorough and effective remediation of Cr(VI) in Cr(VI)-contaminated groundwater aquifer.
